# Supplementary material for: Population Genetics of the Highly Polymorphic RPP8 Gene Family
Source: Genes (Basel). 2019 Sep 8;10(9):691. doi: 10.3390/genes10090691 (PMC6771003; doi:10.3390/genes10090691)
Supplement: Supplementary file 1 [file genes-10-00691-s001.pdf]

Supplementary Figures and Tables

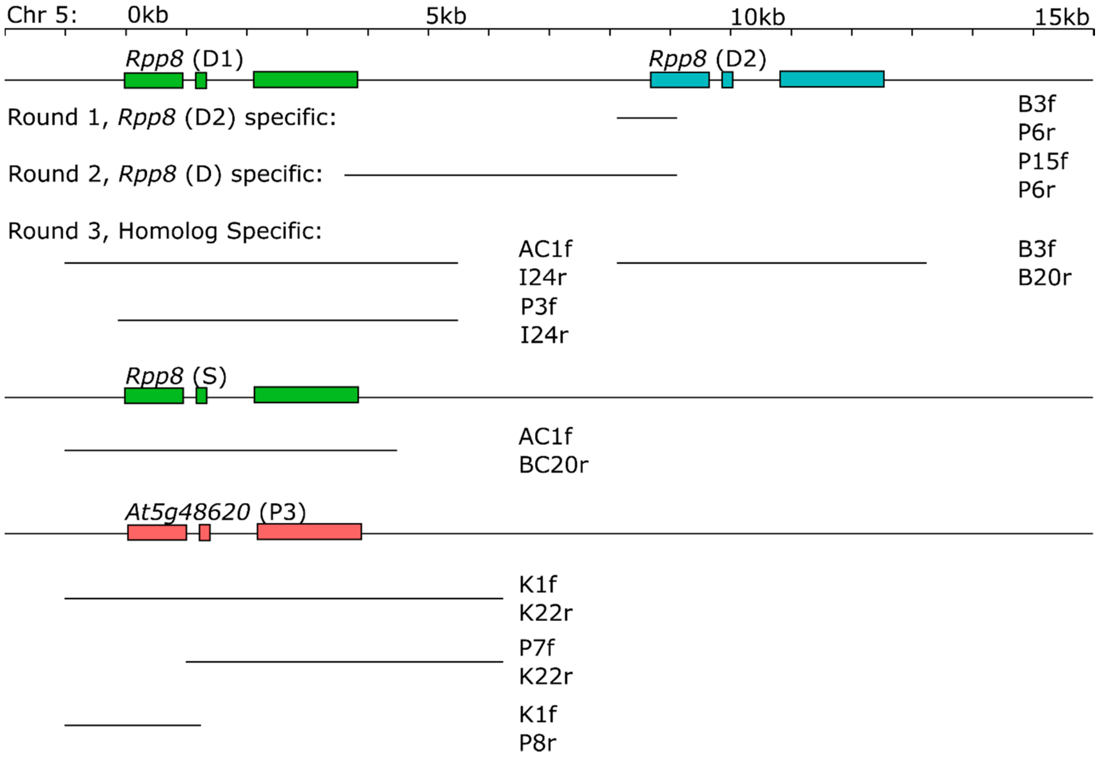

Figure 1. Regions amplified with PCR to sequence *RPP8* alleles in *A. thaliana*. Positions are shown in kilobases, relative to the start codon of the first paralog at that chromosomal location. Exons of *RPP8* loci are shown as boxes. The three rounds of PCR are aligned below the region they amplified.

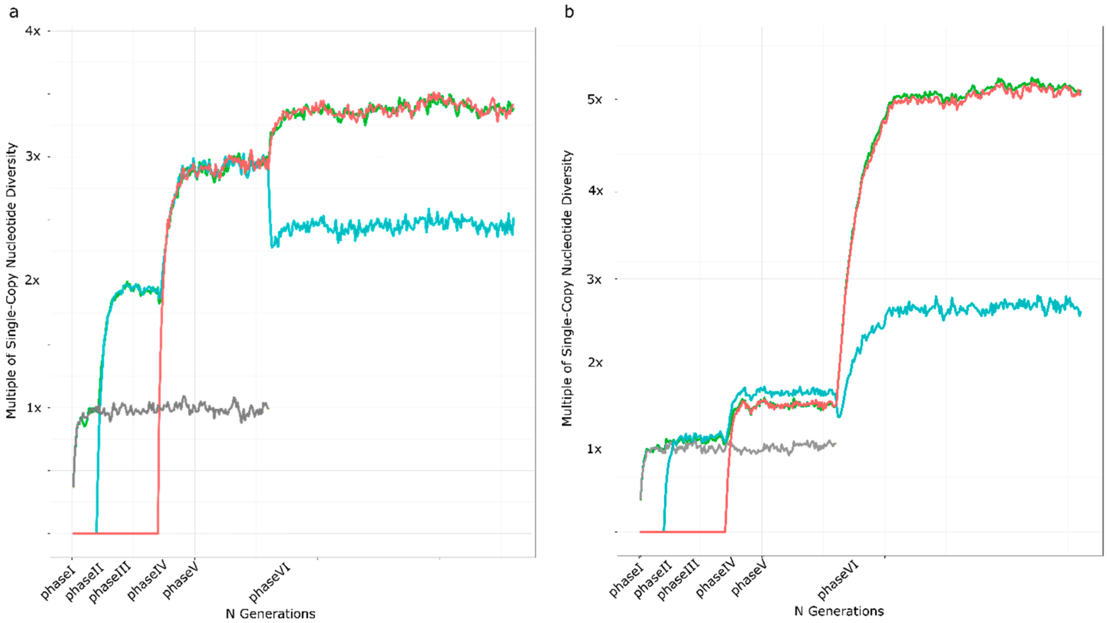

Figure 2. Example extended SeDuS output for the six simulated phases. Lines represent the average of 100 simulations given specific rates of crossover within and between duplicates (R_C, R_{S1}, R_{S2}), and number of IGC events per generation (C). Nucleotide diversities relative to a single copy locus for one copy (grey) and three copy (green, blue, and orange are copies one, two, and three, respectively) systems are shown. **(a)** R = 3.2 for all five chromosomal blocks simulated, equivalent a tandem triplication of a gene the size of *RPP8*; C = 200. **(b)** R_C and R_{S1} of 0.096 for four of five chromosomal blocks simulated; R_{S2} of 60 for the spacer between copy 2 and copy 3 (equivalent to P2 and P3 of *RPP8*); C = 2.

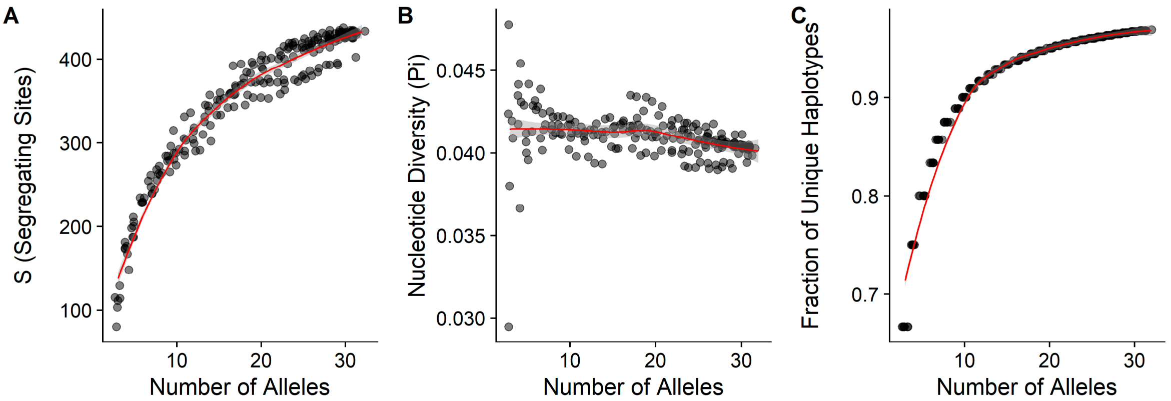

Figure 3. The number of *RPP8* alleles sequenced was sufficient to capture variation in the number of segregating sites **(a)**, nucleotide diversity **(b)**, and the fraction of unique haplotypes **(c)**. Plots show population genetic parameters measured for various subsets of numbers of fully-sequenced alleles used in this study (See Table S1).

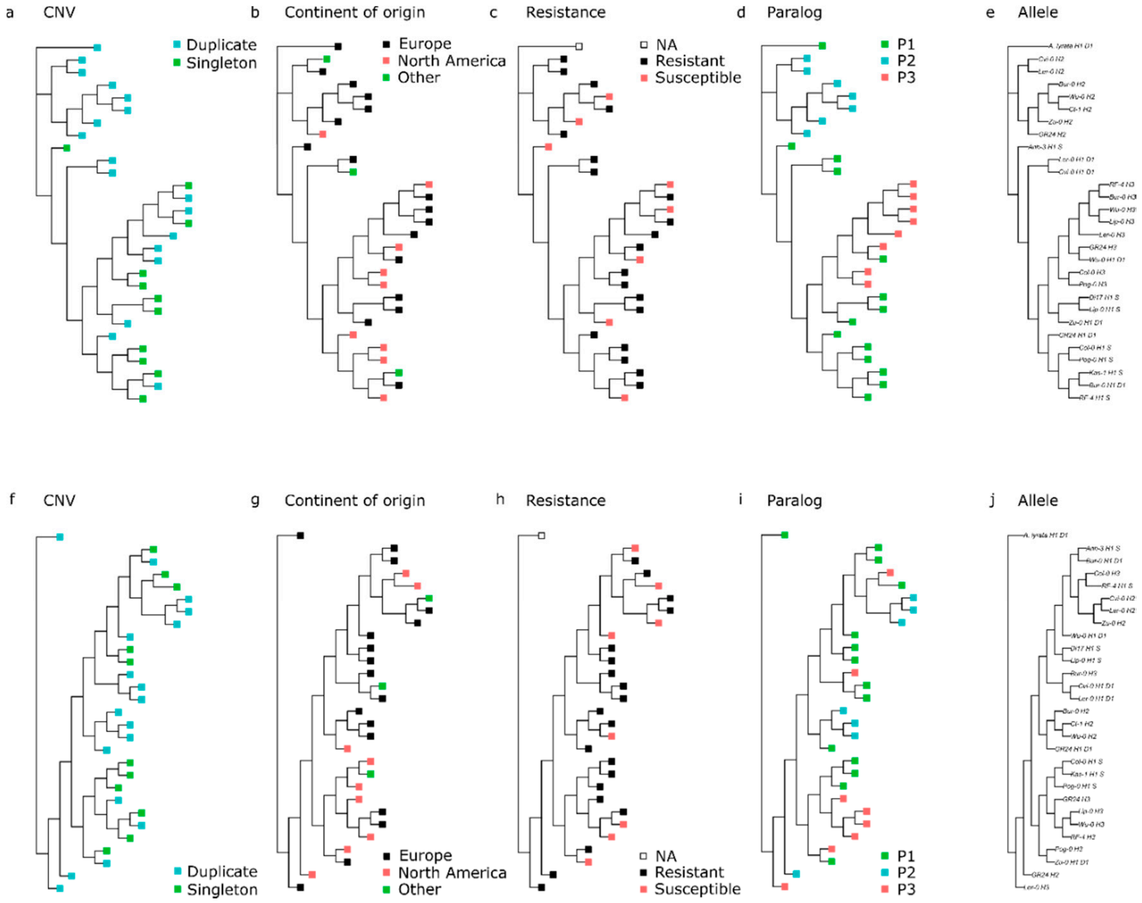

Figure 4. Traits mapped onto **(a-e)** the 1701bp non-leucine rich repeat sequence, with 239 parsimony-informative sites and **(f-j)** the 1019bp leucine-rich repeat sequence, with 236 parsimony-informative sites. **(a,f)** Alleles from accessions with duplicated (cyan) and singleton (green) variants of *RPP8*. **(b,g)** Alleles from accessions European (black), North American (orange) and other (green) locations of origin for the accessions sequenced for paralogs of *RPP8*. **(c,h)** Alleles from accessions which were resistant (black) and susceptible (orange) to *Hyaloperonospora arabidopsidis*. **(d,i)** Allele locations in the genome – P1 (green), P2 (cyan), and P3 (orange). **(e,j)** Accession names and allele locations for each allele in the phylogenies in **(a-d)** and **(f-i)**, respectively.

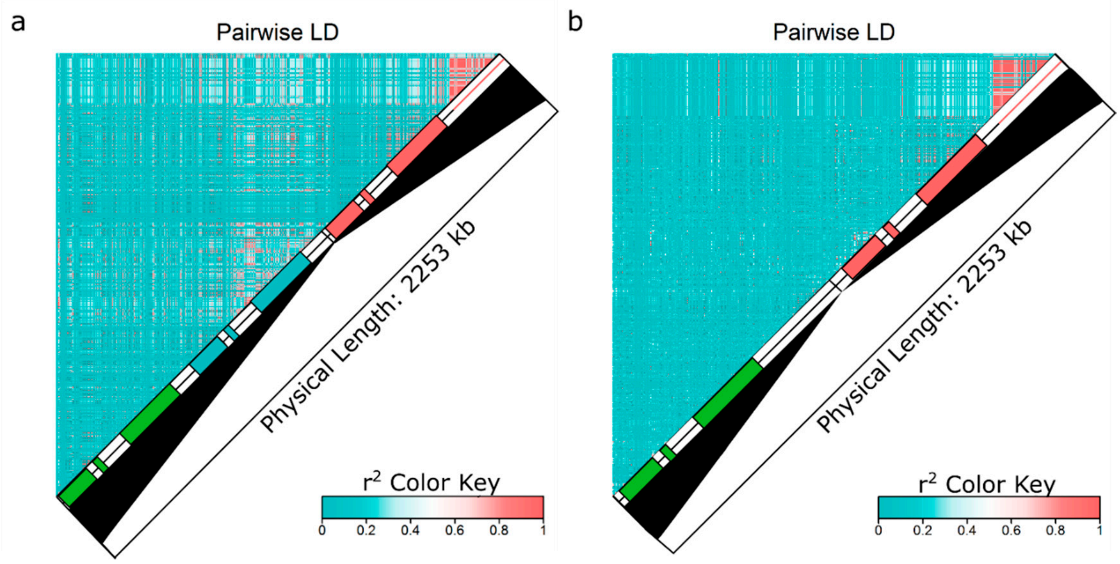

Figure 5. Linkage disequilibrium (LD) within and between the three members of the *RPP8* gene family. Green, blue, and orange boxes represent positions of exons of P1, P2, and P3, respectively; orange line indicates the 3′ region of *At5g48620* with no homology with other members of the *RPP8* gene family. **(a)** LD within and between D1 and D2 variants and P3. **(b)** LD within and between P1 and P3 variants.

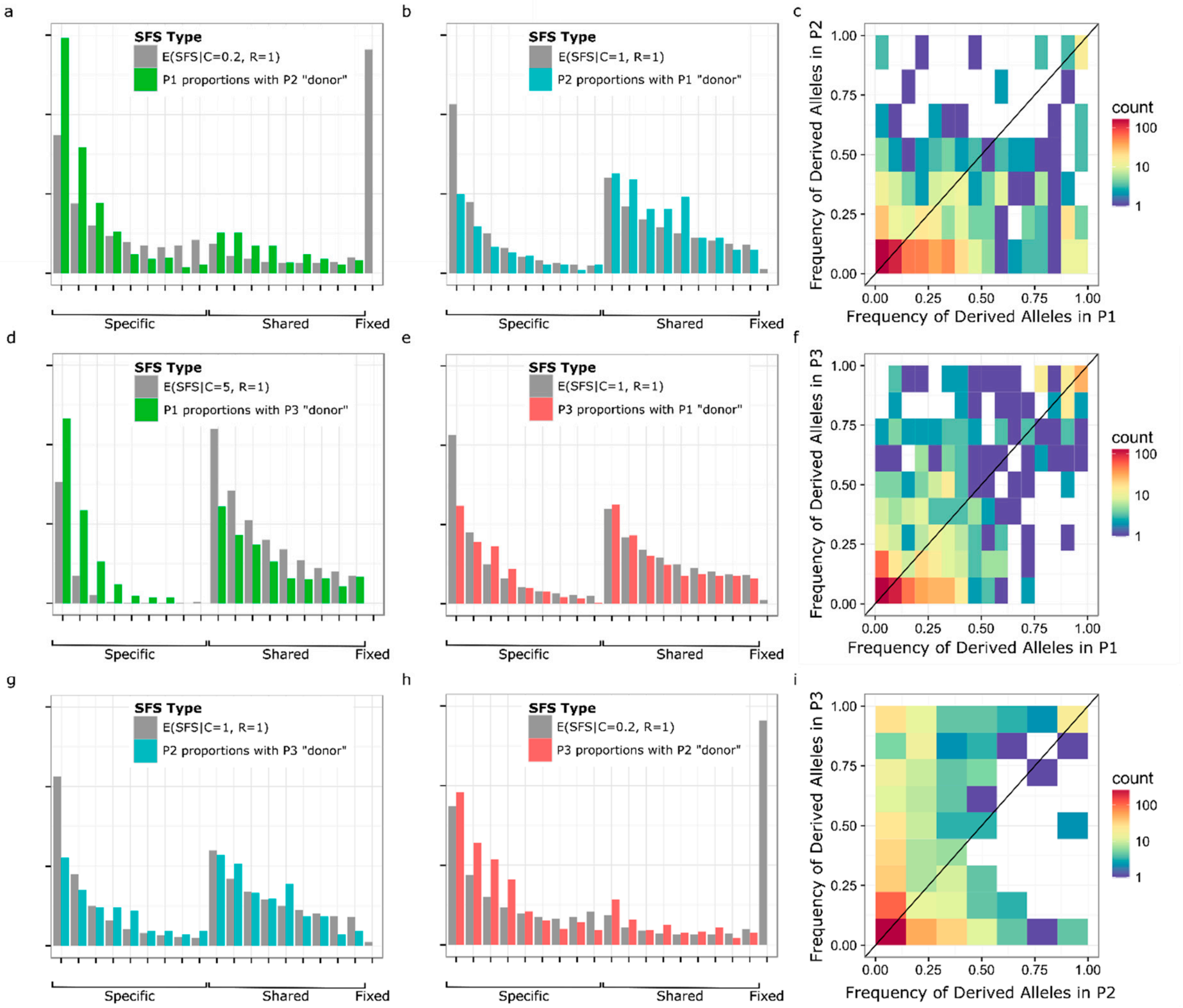

Figure 6. Site frequency spectra (SFS) between pairs of *RPP8* paralogs compared to the most similar expected SFS from [41]. Green, blue, and orange represent observed SFS in paralogs P1, P2, and P3, respectively, while grey represents the expected SFS. The three expected SFS with intergenic gene exchange rates of 0.2,1, and 5 exchanges per generation can be seen in panels a, b, and d. **(a-b)** SFS between P1 and P2 showing frequencies of derived alleles in P1 and P2, with the "donor" considered P2 or P1, respectively. **(d-e)** SFS between P1 and P3 showing frequencies of derived alleles in P1 and P3, with the "donor" considered P3 or P1, respectively. **(g-h)** SFS between P2 and P3 showing frequencies of derived alleles in P2 and P3, with the "donor" considered P3 or P3, respectively. **(c,f,i)** Two-dimensional heatmap representations of the data shown in **(a-b)**, **(d-e)**, and **(g-h)**, respectively.

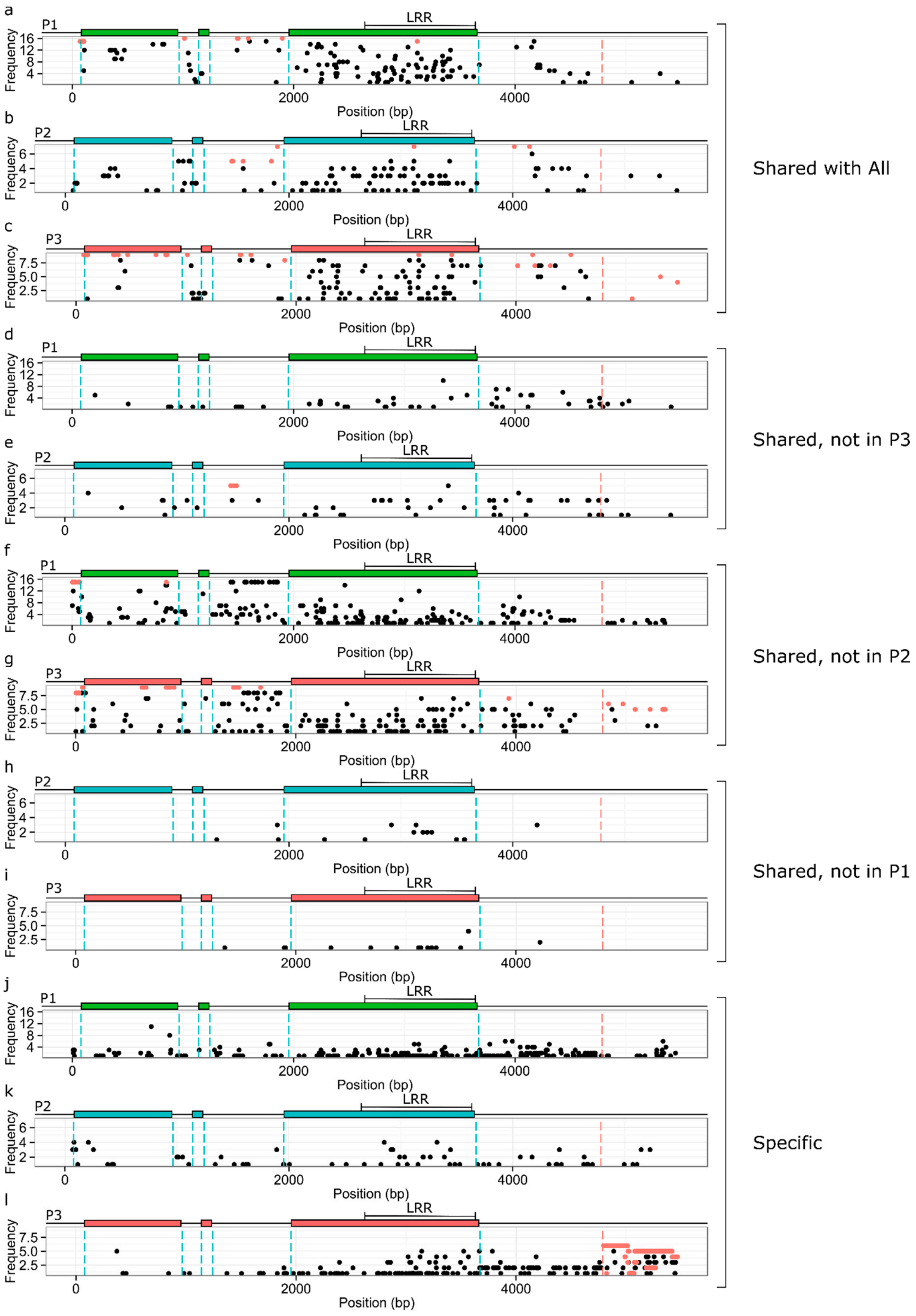

Figure 7. Polymorphism frequencies by site, out of 470 segregating sites within the *RPP8* gene family. Plots show the frequencies of derived SNPs shared with other paralogs or specific to that paralog against the position of the SNP on the sequence. Green, blue, and orange boxes represent positions of exons of paralogs P1, P2, and P3, respectively. Dotted blue lines show the intron and exon boundaries.

Dotted orange line shows the downstream duplication boundary for *At5g48620*. Orange points represent fixed derived alleles. **(a-c)** Shared polymorphisms found in all three members of the *RPP8* gene family; frequencies of each shared SNP in P1, P2, and P3, respectively. **(d-i)** Polymorphisms shared between two *RPP8* paralogs that were not observed in the third. **(d-e)** Polymorphisms shared between P1 and P2; frequencies in P1 and P2, respectively. **(f-g)** Polymorphisms shared between P1 and P3; frequencies in P1 and P3, respectively. **(h-i)** Polymorphisms shared between P2 and P3; frequencies in P2 and P3, respectively. **(j-l)** Polymorphisms specific to each of the three *RPP8* paralogs; SNP frequencies in P1, P2, and P3, respectively.

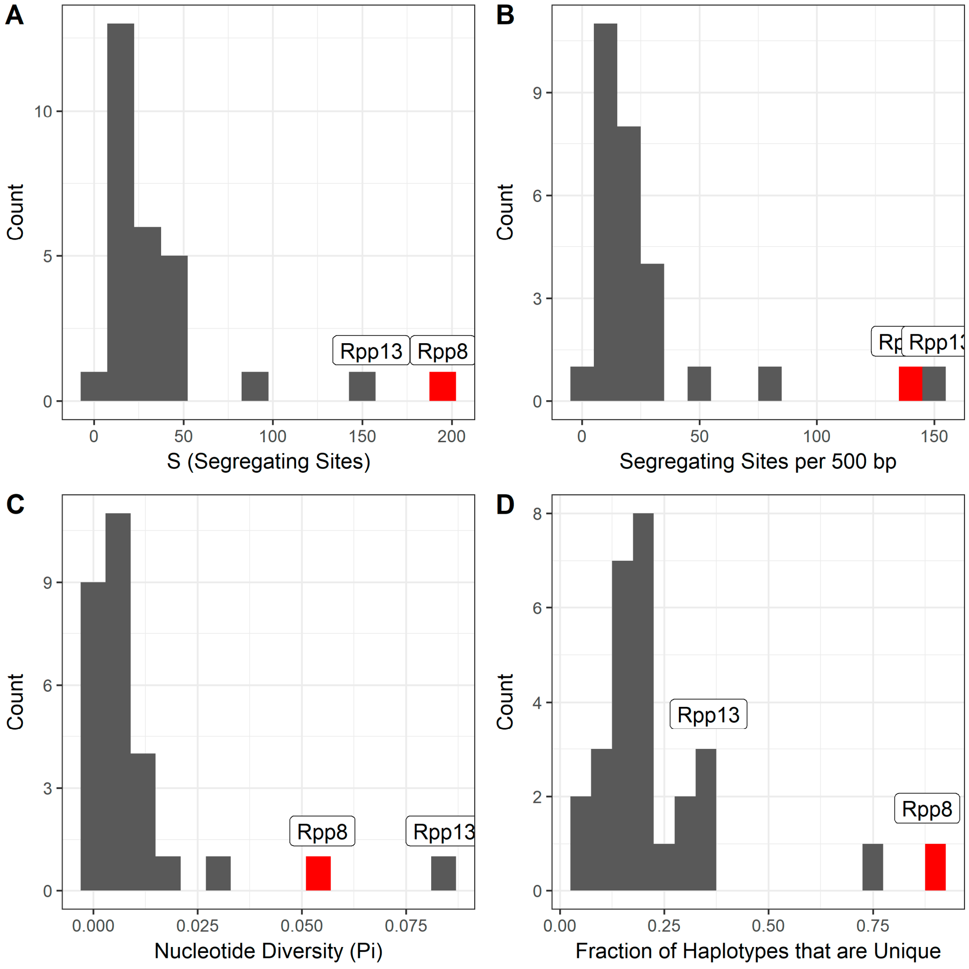

Figure 8. The distributions of population genetic summary statistics were higher for *RPP8* than for singleton NLR genes. Plots show the number of segregating sites and segregating sites per 500 bp **(a,b)**, nucleotide diversity **(c)**, and the fraction of unique haplotypes **(d)**. These population genetic parameters were measured for ~1kb regions of the leucine-rich repeat (LRR) for 50 *RPP8* paralogs. [32] conducted Sanger sequencing on ~1kb regions of the LRR for 56 to 92 accessions and for 27 singleton NLR genes. Information on the accessions compared can be found in Table S1. *RPP13* and *RPP8* are both labeled; *RPP13* is frequently an outlier among singleton NLR genes. *RPP8* values are highlighted in red.

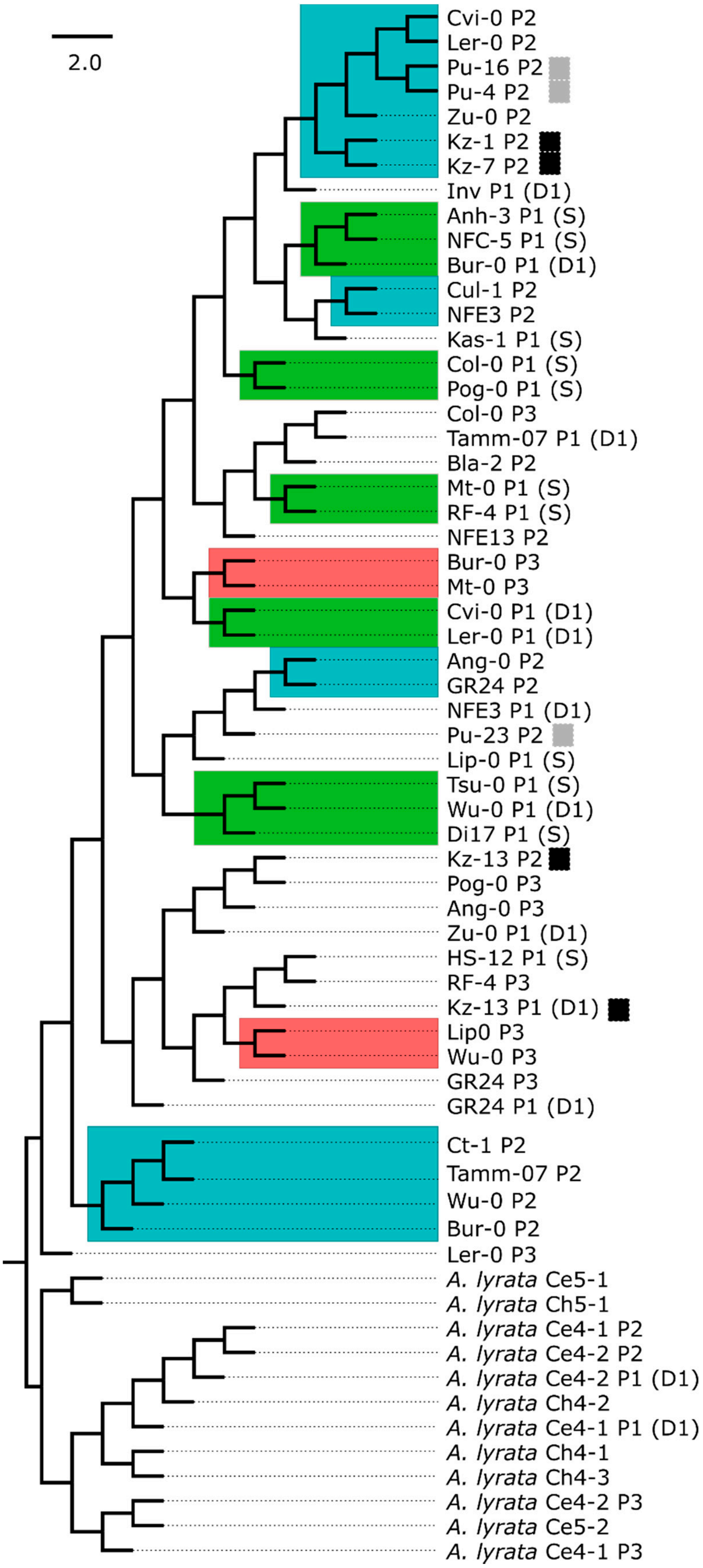

Figure 9. Sequence similarity tree of the 888bp leucine-rich repeat (LRR) sequence obtained for 50 alleles of the *A. thaliana RPP8* gene family and 12 alleles of the *A. lyrata RPP8* gene family. Clades comprised of alleles from one paralog are boxed. Green, blue, and orange boxes represent *RPP8* paralogs P1, P2, and P3, respectively. 228 sites were parsimony-informative. Grey boxes by accession

names represent alleles from the Pu- population in the Czech Republic, and black boxes by accession names represent alleles from the Kz- population in Kazakhstan.

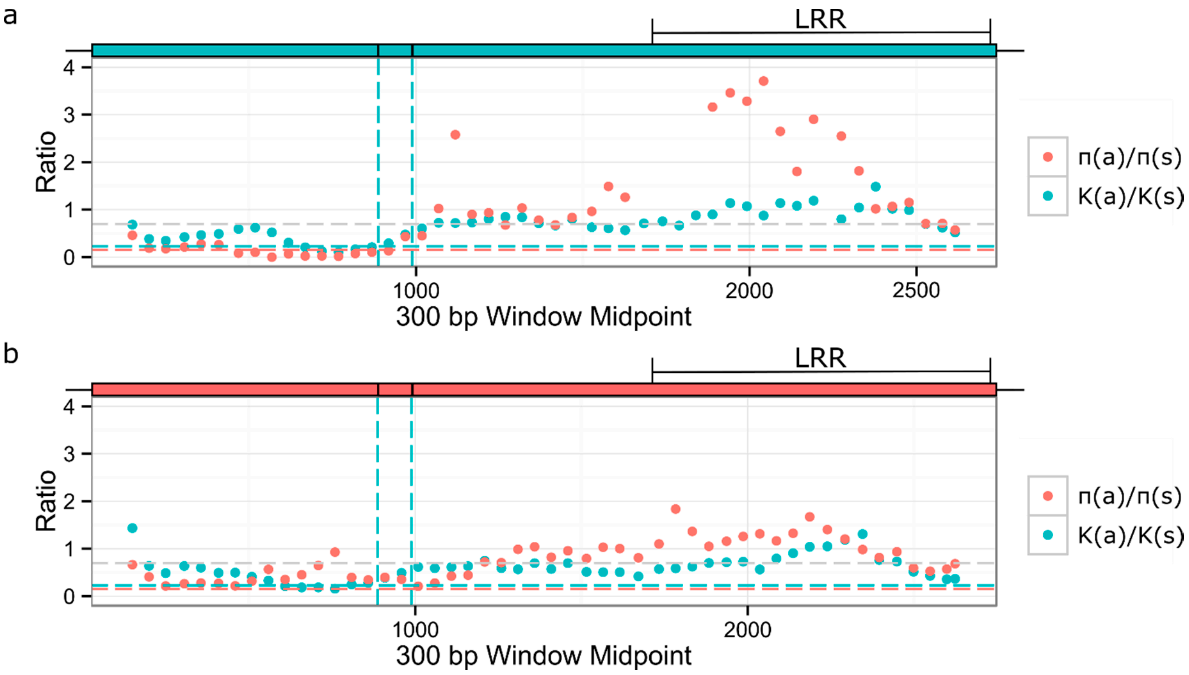

Figure 10. Sliding window analysis of within-species polymorphism and divergence between *A. thaliana* and *A. lyrata* in the coding region for paralogs P2 and P3 of *RPP8*. Blue and orange boxes above the plots represent positions of exons of P2 and P3, respectively. Vertical lines indicate exon boundaries, as shown in the schematic above each plot. The leucine-rich repeat region (LRR) is also indicated. Orange and blue dashed horizontal lines indicate average levels of π_a:π_s and K_a:K_s within *A. thaliana* and between *A. thaliana* and *A. lyrata*; grey dashed line is the 95% right-hand tail for K_a:K_s. **(a)** Paralog P2 at *RPP8*. **(b)** Paralog P3 at *At5g48620*.

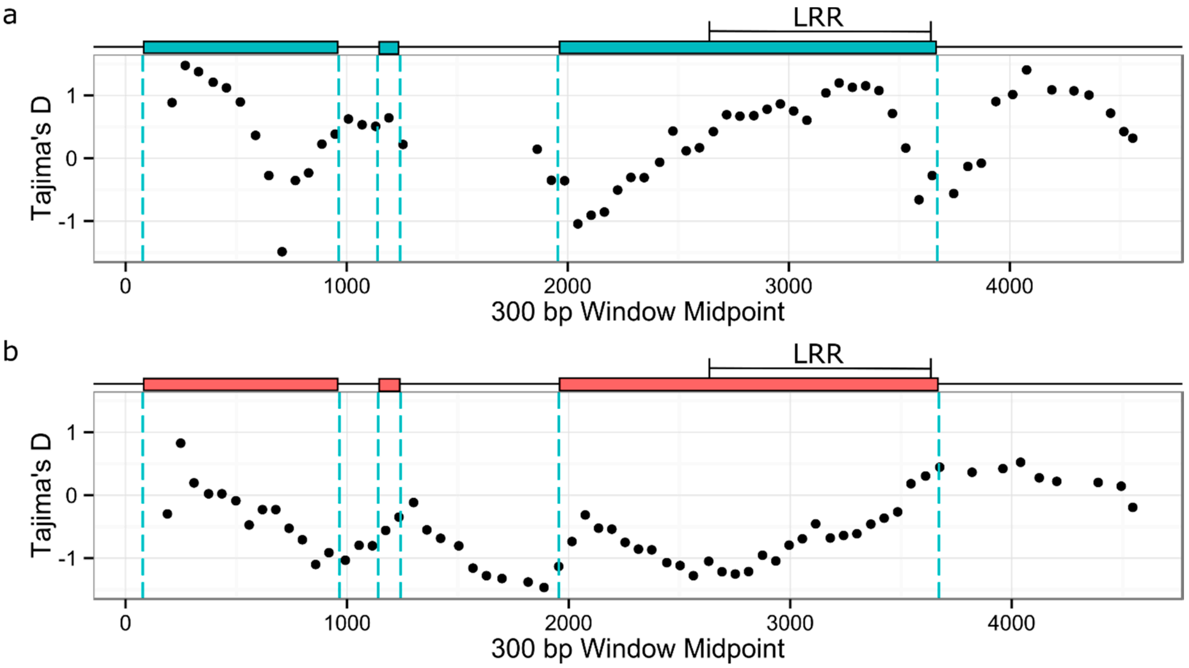

Figure 11. Sliding window analysis of Tajima's D across the sequenced regions for paralogs P2 and P3 of *RPP8*. Blue and orange boxes above the plots represent positions of exons of P2 and P3, respectively. Vertical lines indicate boundaries of coding regions of *RPP8*, as shown in the schematic above each plot. The leucine-rich repeat region (LRR) is also indicated. **(a)** Paralog P2 at *RPP8*. **(b)** Paralog P3 at *At5g48620*.

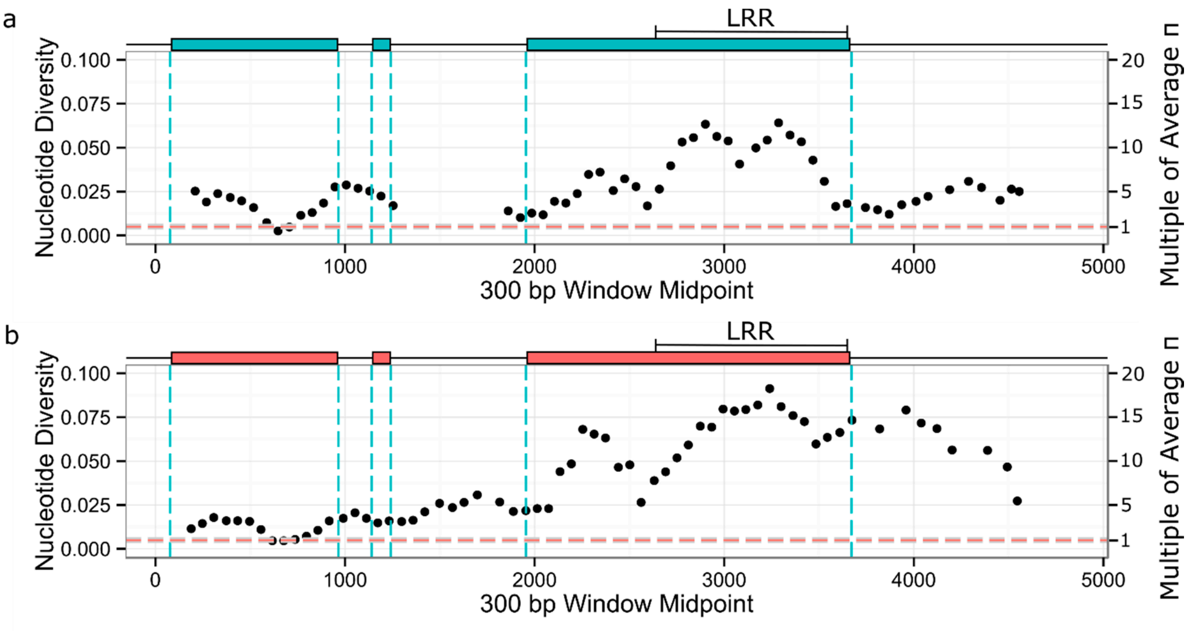

Figure 12. Sliding window analysis of nucleotide diversity across the sequenced regions for paralogs P2 and P3 of *RPP8*. Blue and orange boxes above the plots represent positions of exons of P2 and P3, respectively. Vertical lines indicate boundaries of coding regions of *RPP8*, as shown in the schematic above each plot. The leucine rich repeat region (LRR) is also indicated. The horizontal dashed line indicates the average level of nucleotide diversity within *A. thaliana*; the line width is the confidence interval for average nucleotide diversity. **(a)** Paralog P2 at *RPP8*. **(b)** Paralog P3 at *At5g48620*. **.**

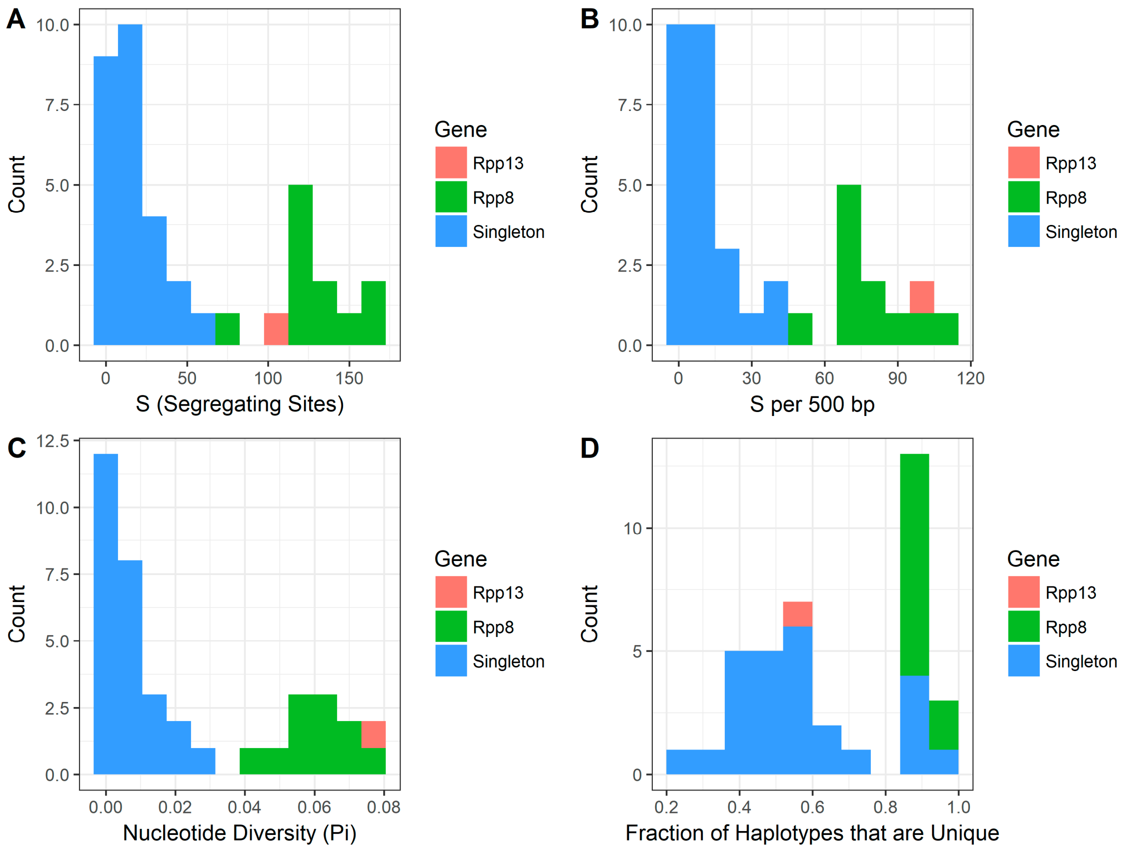

Figure 13. The distributions of population genetic summary statistics were higher for *RPP8* than for singleton NLR genes. Plots show the number of segregating sites and segregating sites per 500 bp **(a-b)**, nucleotide diversity **(c)**, and the fraction of unique haplotypes **(d)**. Parameters were measured for 11 7-11 allele subsets of 20 ~1kb regions of the leucine-rich repeat (LRR) of *RPP8* alleles that also had

singleton NLR genes sequenced in [32]. [32] conducted Sanger sequencing on ~1kb regions of the LRR for 7-11 accessions that were in this study, and sequenced 27 singleton NLR genes. Information on the accessions compared can be found in Table S1. *RPP13* and *RPP8* are both given distinct colors; *RPP13* is frequently an outlier among singleton NLR genes.

Table S1. Genotypes of sampled accessions.

**Species**	**Accession**	**P1**	**P2**	**P3**	**Genotype *RPP8, At5g48620***	***H. arabidopsidis* phenotype**	**Location of Origin**	**Stock Number**	**In Bakker *et al.,* (2006)**
A. thaliana	Bur-0	P; full[a]	P; full[a]	P; full[a]	D[b], P3[c]	R[d]	Ireland	7058	yes
	GR24	P; full	P; full	P; full	D, P3	R	USA		no
	Ler-0	P; full	P; full	P; full	D, P3	R	Germany	7213	yes
	Wu-0	P; full	P; full	P; full	D, P3	S[d]	Germany	7415	no
	Zu-0	P; full	P; full	P[e]	D, P3	S	Switzerland	7417	no
	Cvi-0	P; full	P; full	Δ[e]	D, Δ[e]	R	Cape Verde Islands	8281	yes
	Inv	P; full	P[e]	P	D, P3	/[f]	England		no
	NFE3	P; full	P	P	D, P3	/	England		no
	Kz-13	P; LRR[g]	P; LRR[g]	P	D, P3	/	Kazakhstan	6830	no
	Tamm-07	P; LRR	P; LRR	P	D, P3	/	Finland		yes
	Ct-1	P[e]	P; full	P	D, P3	R	Italy	6910	yes
	Ang-0	P	P; LRR	P; LRR[g]	D, P3	/	Belgium	6992	no
	Bla-2	P	P; LRR	P	D, P3	S	Spain		no
	Cul-1	P	P; LRR	P	D, P3	S	England	5733	no
	Kz-1	P	P; LRR	P	D, P3	/	Kazakhstan	6930	yes
	Kz-7	P	P; LRR	P	D, P3	/	Kazakhstan		no
	NFE13	P	P; LRR	P	D, P3	/	England		no
	Pu-16	P	P; LRR	P	D, P3	/	Czech Republic		no
	Pu-23	P	P; LRR	P	D, P3	/	Czech Republic	8361	yes
	Pu-4	P	P; LRR	P	D, P3	/	Czech Republic		no
	Kz-4	P	P	P	D, P3	/	Kazakhstan		no
	Pu-5	P	P	P	D, P3	/	Czech Republic		no
	Pu-8	P	P	P	D, P3	/	Czech Republic		no
	Col-0	P; full	Δ[e]	P; full	S[b], P3	S	USA	6909	yes
	Lip-0	P; full	Δ	P; full	S, P3	R	Poland	8325	no
	Mt-0	P; full	Δ	P; full	S, P3	/	Libya	6939	yes
	Pog-0	P; full	Δ	P; full	S, P3	R	Canada	7306	No
	RF-4	P; full	Δ	P; full	S, P3	S	USA		no
	Anh-3	P; full	Δ	P	S, P3	/	Germany		no
	Kas-1	P; full	Δ	P	S, P3	R	India	7183	yes
	Di17	P; full	Δ	/[g]	S, /[g]	R	France		no
	HS-12	P; LRR	Δ	P	S, P3	/	USA		no
	NFC-5	P; LRR	Δ	P	S, P3	/	England		no
	Tsu-0	P; LRR	Δ	P	S, P3	S	Japan	7373	yes
	AB-27	P	Δ	P	S, P3	/	USA		no
	FM-15	P	Δ	P	S, P3	/	USA		no

	UP-14	P	Δ	P	S, P3	/	USA	no
A. lyrata	CE (3 ind.)	P; full	P; full	P; full	D, P3	/	USA	no
	CH (4 ind.)	P; full	P; full	P; full	D, P3	/	USA	no

[a]P; full represents an accession with the full coding sequence for the *RPP8* paralog at that genomic location (Sequences of Ler-0, Col-0 and Di17 came from GeneBank). This sequence data is used in Figures 1-3, Tables 1-5, Fig S3-S5 and S6-S8, and Table S7.

[b]D represents the chromosomal haplotype carrying *RPP8* tandem variants (D1,D2); S represents single copy *RPP8* variants.

[c]P3 represents the chromosomal haplotype carrying at least one copy of *At5g48620*.

[d]R represents accessions resistant *to H. arabidopsidis*; S represents susceptible accessions.

[e]P represents the presence of a paralog at this genomic location; Δ represents the absence of a paralog.

[f]/ designates no genotyping and/or no phenotyping for this paralog or accession.

[g]P; LRR represents sequencing for just an 888bp region comprising 12 of 14 leucine-rich repeats for that paralog. This sequence data, along with the 888bp region from the full sequences, was used for Tables 1 and 4 and Fig S5.

Table S2. Target location and sequence of primers that amplified *RPP8* paralogs.

Primer[1]	Location: relative to start codon of gene	Sequence (5′ -3′)
B3f	6826 –447	GGGAAGAAGATGCCTGGGAGTGA
AC1f[2]	-1001 -982	GATCAATGCAGCGAAGGTGTA
BC20r	11798 4525 4570	CACCAATCTGAACTGAAACCTAC
I24r	5481	AGTTTTAGTTTTGATGTATGTG
P3f	-44, 7229 -44 -44	GTTCTTGTACTGGTTCATCGTAG
P5f	452, 7715 443 452	AGGGAGATCCGACAAACGTAT
P6r	534, 7797 525 534	TGAACATCATTCTCCACCAAA
P7f	975, 8236 966 975	CCCTAGCATGAGAAACACAAA
P8r	1038, 8298 1026 1038	CAGCATGTATCCCAACACCTT
P9f	1327, 8593 1321 1327	CTAAAAACGTATGGTAATCCA
P10r	2150, 9415 2141 2150	GATCCATCGTAAATCCCTTCT
P11f	2524, 9800 2527 2530	TTGCTCAGGGTGTTGGATCTT
P12r	2641, 9917 2644 2647	GTTCCGCATAGTAGAAGGTAG
P15f	3473, 10748 3476 3479	ACAAAGTCCAACACATTCCCG
P16r	3648, 10924 3650 3655	CTTCTTGGTCTTTCCTGCATC
P20r	4441, 11687 4414 4459	TGTTGTTACTAGAAGGCATGGTC
K1f		
K22r		

[1] The locations and sequences of primers are based on Ler-0 sequence (accession no. AF089710) for gene D2 & *At5g48620*, or based on Col (AF089711) in GeneBank;
[2] Sequence of Primer AC1 came from accession AB025638 in GeneBank.

Table S3. Parameter sets for Figure 4a and 4d, extended SeDuS runs varying the distance between the second and third copy of the simulated gene family.

	~2 kb	~20 kb	~200 kb	~2 Mb	~20 Mb	~200 Mb	~2 Gb	~20 Gb
N	100	100	100	100	100	100	100	100
R_C	3.2	3.2	3.2	3.2	3.2	3.2	3.2	3.2
R_{S1}	2.4	2.4	2.4	2.4	2.4	2.4	2.4	2.4
R_{S2}	1.6E00	1.6E01	1.6E02	1.6E03	1.6E04	1.6E05	1.6E06	1.6E07
C	8.4	8.4	8.4	8.4	8.4	8.4	8.4	8.4
s	0.97	0.97	0.97	0.97	0.97	0.97	0.97	0.97
μ	0.001	0.001	0.001	0.001	0.001	0.001	0.001	0.001
Exchange type	unequal	unequal	unequal	unequal	unequal	unequal	unequal	unequal

Table S4. Parameter sets for Figure 4b and 4e, extended SeDuS runs varying the total IGC rate within the simulated gene family.

	C = 0.2	C = 1	C = 2	C = 5	C = 8.4	C = 20	C = 200	C = 2000
N	100	100	100	100	100	100	100	100
R_C	3.2	3.2	3.2	3.2	3.2	3.2	3.2	3.2
R_{S1}	2.4	2.4	2.4	2.4	2.4	2.4	2.4	2.4
R_{S2}	1.6E04	1.6E04	1.6E04	1.6E04	1.6E04	1.6E04	1.6E04	1.6E04
C	0.2	1	2	5	8.4	20	200	2000
s	0.97	0.97	0.97	0.97	0.97	0.97	0.97	0.97
μ	0.001	0.001	0.001	0.001	0.001	0.001	0.001	0.001

Exchange type	equal	equal	equal	equal	equal	equal	equal	equal

Table S5. Parameter sets for Figure 4c and 4f, extended SeDuS runs varying both total IGC rate and IGC directionality within the simulated gene family.

	***C* = 0.2**	***C* = 0.2**	***C* = 2**	***C* = 2**	***C* = 20**	***C* = 20**	***C* = 200**	***C* = 200**
N	100	100	100	100	100	100	100	100
R_C	3.2	3.2	3.2	3.2	3.2	3.2	3.2	3.2
R_{S1}	2.4	2.4	2.4	2.4	2.4	2.4	2.4	2.4
R_{S2}	1.6E04	1.6E04	1.6E04	1.6E04	1.6E04	1.6E04	1.6E04	1.6E04
C	0.2	0.2	2	2	20	20	200	200
s	0.97	0.97	0.97	0.97	0.97	0.97	0.97	0.97
μ	0.001	0.001	0.001	0.001	0.001	0.001	0.001	0.001
Exchange type	equal	unequal	equal	unequal	equal	unequal	equal	unequal

Table S6. Parameter sets for Figure 5, varying the fraction of individuals that self within the population. *C* was 0.2 for Figure 5a and 5d, 8.4 for 5b and e, and 200 for 5c and f.

	**Outcrossing -> Increasing Selfing**						
N	100	100	100	100	100	100	100
R_C	106.56	105.6	96	80	53.333	26.67	10.67
R_{S1}	79.92	79.2	72	60	40	20	8
R_{S2}	59940	59400	54000	45000	30000	15000	6000
C	0.2; 8.4; 200	0.2; 8.4; 200	0.2; 8.4; 200	0.2; 8.4; 200	0.2; 8.4; 200	0.2; 8.4; 200	0.2; 8.4; 200
s	0.001	0.01	0.10	0.25	0.50	0.75	0.90
μ	0.001	0.001	0.001	0.001	0.001	0.001	0.001
Exchange type	unequal	unequal	unequal	unequal	unequal	unequal	unequal
	**Outcrossing -> Increasing Selfing**						
N	100	100	100	100	100	100	
R_C	8.53	5.33	3.2	2.13	1.07	0.1066	
R_{S1}	6.40	2.13	2.4	1.60	0.80	0.08	
R_{S2}	4800	3000	1800	1200	600	60	
C	0.2; 8.4; 200	0.2; 8.4; 200	0.2; 8.4; 200	0.2; 8.4; 200	0.2; 8.4; 200	0.2; 8.4; 200	
s	0.92	0.95	0.97	0.98	0.99	0.999	
μ	0.001	0.001	0.001	0.001	0.001	0.001	
Exchange type	unequal	unequal	unequal	unequal	unequal	unequal	

Table S7. R^2 values for linear models of the correlation in SNP frequencies for shared polymorphisms in the sequenced duplicated region for comparisons between locus X, rows, and Y, columns.

**X / Y**	**D1**	**D2**	**P3**
S	R^2 = **0.673****	R^2 = **0.0699****	R^2 = **0.506****
D1		R^2 = 0.0458*	R^2 = **0.473****
D2			R^2 = 0.04313*

* represents correlations significant at the < 0.01 level;

** and bolded values represent correlations significant at the < 0.001 level.
